# Supplementary figures and images for: UBR5 in Tumor Biology: Exploring Mechanisms of Immune Regulation and Possible Therapeutic Implications in MPNST
Source: Cancers (Basel). 2025 Jan 7;17(2):161. doi: 10.3390/cancers17020161 (PMC11764400; doi:10.3390/cancers17020161)

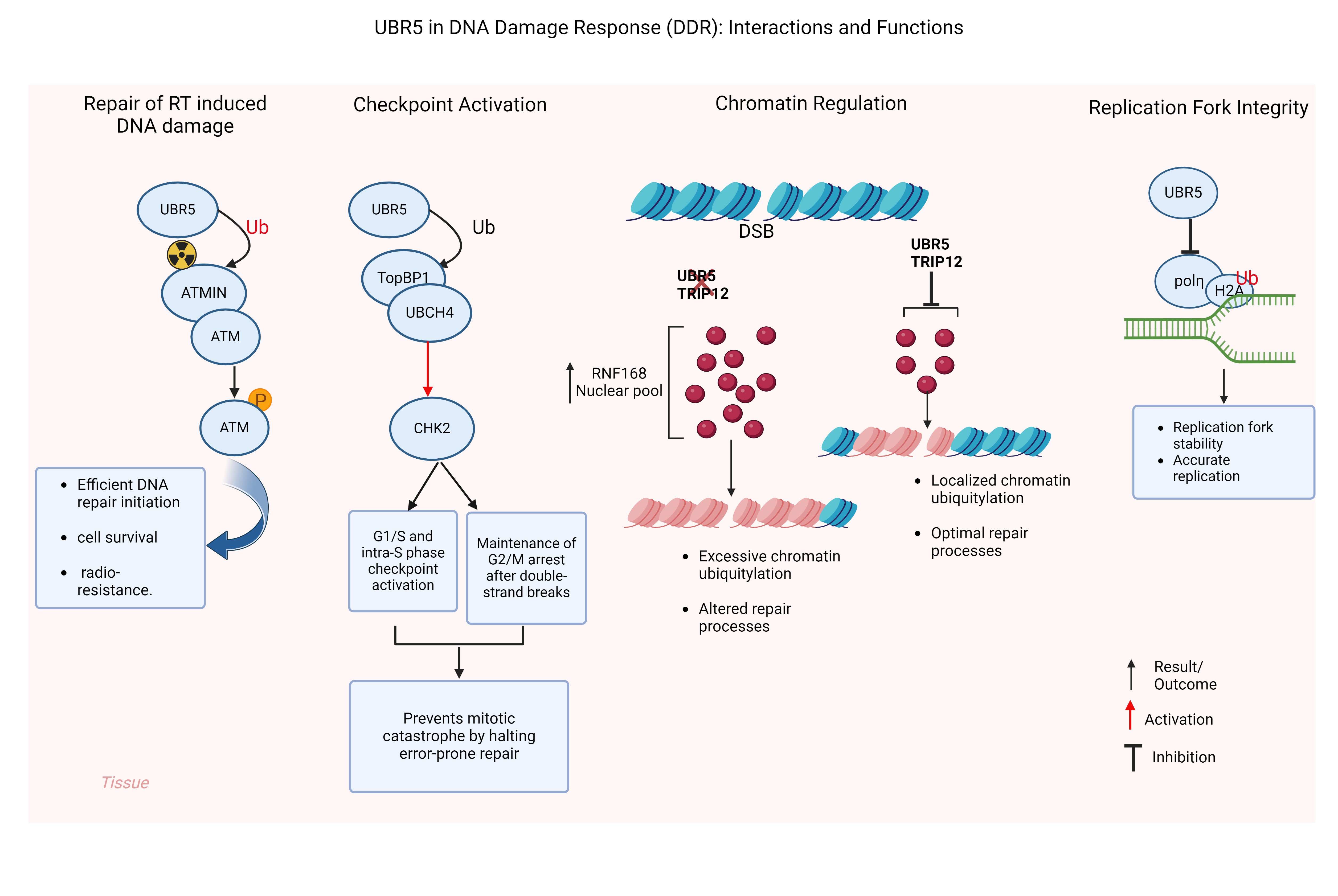

Supplement: Supplementary file 1 [file cancers-17-00161-s001.zip › cancers-3359471-supplementary Figure S1.png]
